# Risk factors for infection and disease in child contacts of multidrug-resistant tuberculosis: a cross-sectional study

**DOI:** 10.1186/1471-2334-13-392

**Published:** 2013-08-26

**Authors:** James A Seddon, Anneke C Hesseling, Peter Godfrey-Faussett, Katherine Fielding, H Simon Schaaf

**Affiliations:** 1 Desmond Tutu TB Centre, Department of Paediatrics and Child Health, Faculty of Medicine and Health Sciences, Stellenbosch University, Clinical Building, Room 0085, P. O. Box 19063, Tygerberg, South Africa; 2 Department of Clinical Research, Faculty of Infectious and Tropical Diseases, London School of Hygiene and Tropical Medicine, London, UK; 3 Department of Infectious Diseases Epidemiology, Faculty of Epidemiology and Population Health, London School of Hygiene and Tropical Medicine, London, UK; 4 Tygerberg Children’s Hospital, Tygerberg, South Africa

**Keywords:** Pediatric, Infection, Disease, Drug-resistant, Tuberculosis, Children

## Abstract

**Background:**

Young children exposed to *Mycobacterium tuberculosis* have a high risk of disease progression following infection. This study aimed to determine risk factors for *M. tuberculosis* infection and disease in children following exposure to adults with multidrug-resistant (MDR) tuberculosis (TB).

**Methods:**

Cross-sectional study; all children aged < 5 years, routinely referred per local guidelines to the provincial specialist MDR-TB clinic, Western Cape Province, South Africa, following identification as contacts of adult MDR-TB source cases, were eligible for enrolment from May 2010 through April 2011. Demographic, clinical and social characteristics were collected. All children underwent HIV and tuberculin skin testing.

**Results:**

Of 228 children enrolled (median age: 30 months), 102 (44.7%) were classified as infected. Of these, 15 (14.7%) had TB disease at enrolment. Of 217 children tested for HIV, 8 (3.7%) were positive. In adjusted analysis, child’s age (AOR: 1.43; 95% CI: 1.13-1.91; p = 0.002) and previous TB treatment history (AOR: 2.51; 95% CI: 1.22-5.17; p = 0.01) were independent risk factors for infection. Increasing age of the MDR-TB source case (AOR: 0.67; 95% CI: 0.45-1.00; p = 0.05) was protective and source case alcohol use (AOR: 2.59; 95% CI: 1.29-5.22; p = 0.007) was associated with increased odds of infection in adjusted analysis. Decreasing age of the child (p = 0.01) and positive HIV status (AOR: 25.3; 95% CI: 1.63-393; p = 0.01) were associated with prevalent TB disease.

**Conclusion:**

A high proportion of children exposed to MDR-TB are infected or diseased. Early contact tracing might provide opportunities to prevent the progression to TB disease in children identified as having been exposed to MDR-TB.

## Background

The development of tuberculosis (TB) requires exposure to *Mycobacterium tuberculosis*, subsequent infection, and finally, progression to disease [1]. The risk of moving from one state to the next is determined by multiple microbiological, immunological, social and cultural factors [1]. The diagnosis of *M. tuberculosis* infection and disease in children is challenging as available tests have limitations. The tuberculin skin test (TST) and commercial interferon-gamma release assays (IGRAs) have limited sensitivity in detecting *M. tuberculosis* infection compared to a reference standard of either an *M. tuberculosis* exposure gradient or confirmed disease [2]. Due to the paucibacillary nature of pediatric TB, together with challenges in obtaining clinical samples, microbiologically confirmed TB is obtained in less than 30% of children with radiological/clinical evidence of intra-thoracic disease, even when sputum induction and a referral laboratory are available [3]. The diagnosis of TB relies on a constellation of history, examination, immunology, radiology and bacteriology, all with limited sensitivity and specificity [4]. In the absence of accurate tests of infection and disease, it is important to understand the epidemiology of each of the exposure, infection and disease stages, and risk factors determining progression between each stage. If these factors are identified, interventions can be targeted to those at the highest risk of progression to the next stage. As childhood TB results from recent infection and subsequent disease development, children serve as a sentinel marker of ongoing *M. tuberculosis* transmission and failing TB control within a community [5]. Identifying risk factors for infection and disease in children is therefore important in understanding the broader epidemic.

For children exposed to drug-susceptible TB, the risk of becoming infected depends on the infectiousness of the source case, the intensity and duration of interaction between the source case and child contact, and the host immune response [1,6]. The influence of mycobacterial strain on risk of infection is unclear [7]. Source cases with extensive lung involvement are more infectious than those with fewer affected zones on chest radiography [8-10]. Children in contact with sputum smear-positive source cases are more likely to become infected than those in contact with sputum smear-negative disease [10,11], with greater bacillary loads increasing infectivity [10,12]. Although HIV-positive adults with TB are more likely to be sputum smear-negative than HIV-negative individuals [13], it does not appear that they are less infectious [14,15], possibly indicating HIV-related epidemiological factors unique to HIV-affected households. Source cases who live in close physical proximity to child contacts are more likely to infect children, and the greater the daily interaction and the longer the duration of interaction, the greater the risk of infection [16,17]. The duration of cough in the source case also affects transmission risk [18]. Close relatives are more likely to infect child contacts than more distant relatives, and sleeping in the same room increases infection risk [8-10,16,19-21]. The presence of smokers in the house has been shown to increase *M. tuberculosis* transmission [11,16,22,23], as does crowding [16] and poor household ventilation [24].

The risk of progression to TB disease in a child infected with *M. tuberculosis* is determined by the virulence of the mycobacteria [25], the mycobacterial load [26], age [27] and host immune response [28]. From studies prior to the chemotherapy era, it is has been determined that infected infants (i.e. <12 months) have a 50% life-time risk of progression to disease following infection, with the risk decreasing with increasing age [6,27]. Young children also have an increased risk of severe forms of disease such as miliary TB and TB meningitis [27]. HIV-positive adults with *M. tuberculosis* infection have a 7-10% risk of developing TB each year in the absence of preventive therapy [29,30]. HIV-positive infants are more than twenty times as likely to develop TB as those who are HIV-negative [31] and malnourished children are more likely to progress to TB than those better nourished [11].

Although surveillance data are limited, the World Health Organization (WHO) estimated that there were 650,000 prevalent cases of multidrug-resistant (MDR)-TB worldwide in 2010 [32]. MDR-TB is defined as *M. tuberculosis* resistant to rifampin and isoniazid [33]. A previous MDR-TB contact study has demonstrated nearly two child contacts less than five years of age for each MDR-TB source case in South Africa, a high TB-burden setting [34], suggesting that large numbers of children are exposed to MDR-TB in such contexts. Determining which children are at the highest risk of becoming infected and of developing disease would allow informed health planning and targeted use of available healthcare resources. A cohort study design, where children are followed from the time of exposure, allows for the monitoring of transition from exposure to infection, and from infection to disease, over time. However, cross-sectional study designs, whereby children are identified who have exposure but no infection, exposure and infection and those who present with TB disease, also allow for an assessment of risk factors for each state. We aimed to determine risk factors for the transition from exposure to infection, and from infection to disease in child MDR-contacts, using a cross-sectional study design.

## Methods

### Setting and patient population

The overall TB disease notification rate in the Western Cape province of South Africa was 976/100,000 in 2009 [35]; with an incidence among children less than 13 years found to be over 400/100,000 in 2003–2004 [36]. The annual rate of TB infection among school children in Cape Town is about 4%, suggesting very high rates of transmission [37,38]. 8.9% of children with culture-confirmed TB at one of two regional referral hospitals, Tygerberg Children’s Hospital (TCH), had MDR-TB during 2007–2009 [39]. Cape Town is comprised of Black (mainly of the Xhosa ethnic group), White (mainly of European ancestry), Indian and Colored (a heterogeneous ethnic group of mixed ancestry) populations. All children evaluated at TCH or community outreach specialist pediatric TB clinics during May 2010 through April 2011, were eligible if they were less than five years old, had been in significant contact with an infectious (sputum smear or culture positive) pulmonary MDR-TB source case within the preceding six months and had an available TST result. Significant exposure was defined as living with or having regular daily interaction with the MDR-TB source case. Children were recruited following written, informed consent from the parent/caregiver. The study was approved by the Stellenbosch University and London School of Hygiene and Tropical Medicine Ethical Committees, as well as the local health authorities.

### Standard of care

Following the diagnosis of MDR-TB in an adult, contact tracing was carried out by the local clinic team to identify vulnerable children. Children exposed to infectious MDR-TB cases and those with suspected MDR-TB disease were referred to a specialist pediatric MDR-TB clinic at TCH or community outreach clinics attended by the same specialists. HIV testing is routinely performed following informed consent from the parent or legal guardian using ELISA in children older than 18 months or DNA PCR if younger or breast-fed. Combination antiretroviral therapy (cART) is routinely initiated in all HIV-positive children following appropriate evaluation. TST was routinely undertaken by local clinic staff, carried out by injecting two tuberculin units intradermally (purified protein derivative RT23, Statens Serum Institute) with results read at 48–72 hours. All children were assessed using TB symptom screening, clinical evaluation and chest radiograph (both antero-posterior and lateral). Children suspected of having TB disease (clinical or radiological evidence) underwent microbiological investigation. Sputum samples, gastric aspirates or biopsies from extra-pulmonary sites were sent for liquid-based culture (MGIT 960 system; Becton Dickinson, Sparks, MD, USA) and drug susceptibility testing (DST) using a commercial Line Probe Assay (GenoType® MTBDR*plus*; Hain Lifescience, Nehren, Germany). They were then started on a treatment regimen tailored to the DST of the source case, or, if disease was bacteriologically confirmed, that of the child. As per local program guidelines, children found to have no symptoms, signs or radiology suggestive of TB disease, were treated with a three-drug preventive therapy regimen of ofloxacin (15-20 mg/kg), ethambutol (20-25 mg/kg daily) and high-dose isoniazid (15-20 mg/kg daily) for six months with regular follow up for one year to monitor for incident disease [40].

### Data collection and classification

This study employed a cross-sectional design. Following informed consent, families were interviewed by the study team and data collected regarding the demographic profile and clinical condition of the child. Information on the source case was collected both from the attending families and subsequently from the provincial TB register. The nature and intensity of the interaction between the source case and child, as reported by the parent/caregiver, was documented as well as the duration of exposure. Children were classified as uninfected or infected, and if infected, as having TB disease or not. While we aimed to assess risk factors for prevalent TB disease, confirmation of diagnosis depended on radiological and bacteriological investigations, in some instances requiring a number of weeks for liquid culture results. Standard research definitions were applied to classify TB disease [41]; children with either confirmed and probable disease were included. Infection was classified as having a positive TST; infection was also assumed in the presence of TB disease. A transverse TST diameter of ≥10 mm was considered positive in HIV-negative and ≥5 mm in HIV-positive children.

### Statistical analysis

Data were double-entered into a database and checked for entry errors. Logistic regression was used to assess risk factors for (i) *M. tuberculosis* infection and (ii) TB disease (among those with infection). Results are reported as unadjusted and adjusted odds ratios (OR), 95% confidence intervals (CIs) and p-values, calculated using the likelihood ratio test (LRT). For ordinal variables a test for trend and departures from linearity using the LRT was conducted. For models assessing risk factors for infection, exposures were included if they demonstrated a relationship with infection in univariable analysis based on p < 0.05. If two exposures were thought to be co-linear they were not included together in the same model. For models assessing risk factors for disease, variables were only adjusted for the age of the child, given the small number of children with disease. Ages for the child and source case were recorded as ordinal variables, using age bands. Relationships between exposures were assessed using the χ^2^ test. All statistical analyses were conducted using Stata software (version 11; Stata Corp, College Station, TX).

## Results

### Description of cohort

Over the twelve-month study period 377 children were referred to the specialist clinic services as child contacts of MDR-TB source cases. A number of children did not meet the eligibility criteria for the study: the source case did not have MDR-TB (n = 27), the child was older than five years (n = 56), the intensity of contact was not judged to be significant by the clinical team (n = 11) or a TST result was not available in the presence of an asymptomatic child (n = 2). Of 281 children eligible for the study, 228 (81%) were recruited. The remaining 53 children were not brought to the clinic by a parent or legal guardian who could provide informed consent (n = 31), the parents did not consent to the study (n = 3) or the families left the clinic before the research team could speak to them (n = 19). These children all received routine standard of care. The median age of children recruited was 30 months (inter-quartile range [IQR]: 13–43 months); of 217 children tested for HIV, 8 (3.7%) were positive. Of the 228 children, 102 (44.7%) were classified as *M. tuberculosis*-infected. Of the 102 infected, 15 (14.7%) also had TB disease.

### Risk factors for infection

In adjusted analysis, increasing age of the child was associated with increasing odds of infection (adjusted odds ratio (AOR) for one year increase in age: 1.43; 95% CI: 1.13-1.91; p = 0.002); children of Colored ethnicity (compared to children of Xhosa ethnicity) were also more likely to be infected (AOR: 2.51; 95% CI: 1.22-5.17; p = 0.01). Children with a previous treatment history were more likely to be infected in univariable analysis, with the effect reduced after adjustment. See Table 1. Increasing age of the source case was associated with a reduced odds of infection (AOR: 0.67 for an approximate 10 year increase in age; 95% CI: 0.45-1.00; p = 0.05) and reported alcohol use by source case was associated with increased odds of infection (AOR: 2.59; 95% CI: 1.29-5.22; p = 0.007). Before adjustment there was a strong relationship between HIV status in the source case and *M. tuberculosis* infection in the child, with HIV positivity in the source case associated with lower odds of infection. In multivariable analysis this association was reduced (AOR 0.48, 95% CI 0.22-1.04; p = 0.06). See Table 2.

**Table 1:** Risk factors for infection in child contacts of multidrug-resistant tuberculosis: child and household characteristics (n = 228)

**Variable**		**Total**	**Infected**	**Unadjusted OR**	**p-value**	**AOR**	**p-value**^**1**^
		**(n = 228)**	**(row %)**	**(95% CI)**		**(95% CI)**^**1**^	
			**(n = 102)**				
Age of child (n = 224)	<1 year	53	15 (28.3)	1	0.05	1.43 (1.13–1.91)	0.002^2^
	1–2 years	36	15 (41.7)	1.81 (0.74–4.42)			
	2–3 years	51	25 (49.0)	2.44 (1.08–5.48)	0.007^2^		
	3–4 years	48	27 (56.3)	3.26 (1.43–7.44)			
	4–5 years	36	18 (50.0)	2.53 (1.05–6.14)			
Gender (n = 227)	Female	109	46 (42.2)	1	0.43		
	Male	118	56 (47.5)	1.24 (0.73–2.09)			
Ethnicity	Xhosa	101	30 (29.7)	1	<0.001	1	0.01
	Colored	125	70 (56.0)	3.01 (1.69–5.36)		2.51 (1.22–5.17)	
Previous TB treatment	No	207	86 (41.5)	1	0.002	1	0.08
	Yes	21	16 (76.2)	4.50 (1.59–12.8)		2.76 (0.84–9.12)	
HIV status (n = 217)	Negative	209	96 (45.9)	1.0	0.64		
	Positive	8	3 (37.5)	0.71 (0.16–3.03)			
Weight-for-age z-score (n = 218)	More than −1	138	60 (43.5)	1	0.14		
	−1 to −2	47	26 (55.3)	1.61 (0.83–3.13)			
	Less than −2	33	11 (33.3)	0.65 (0.29–1.44)			
BCG scar visible (n = 222)	No	40	14 (35.0)	1	0.14		
	Yes	182	87 (47.8)	1.70 (0.83–3.47)			
Type of residence (n = 217)	Tin shack	33	8 (24.2)	1	0.007		
	Brick House	168	79 (47.0)	2.77 (1.18–6.50)			
	Wendy House	16	11 (68.8)	6.87 (1.83–25.8)			
Number of rooms in house	1–2	61	28 (45.9)	1	0.58		
	3–4	102	42 (41.2)	0.83 (0.44–1.56)			
	>4	65	32 (49.2)	1.14 (0.57–2.30)			
Number of people living in house	≤5 people	109	47 (43.1)	1	0.64		
	>5 people	119	55 (46.2)	1.13 (0.67–1.91)			
Density of people living in house	≤2 people per room	153	72 (47.1)	1	0.31		
	>2 people per room	75	30 (40.0)	0.75 (0.43–1.31)			
Water source (n = 225)	Piped water in residence	176	84 (47.7)	1	0.06		
	Piped water from public source	49	16 (32.7)	0.53 (0.27–1.03)			
Toilet	Flush toilet in house	163	79 (48.5)	1	0.07		
	Other	65	23 (35.4)	0.58 (0.32–1.05)			

^1^Adjusted for ethnicity, age of child, previous TB in the child, age of source case, alcohol use in source case and HIV status of source case, ^2^Test for trend, *AOR* adjusted odds ratio,* CI* confidence interval.

**Table 2:** Risk factors for infection in child contacts of multidrug-resistant tuberculosis: source case and exposure characteristics (n = 228)

**Variable**		**Total**	**Infected**	**OR**	**p-value**	**AOR**	**p-value**^**1**^
		**(n = 228)**	**(row %)**	**(95% CI)**		**(95% CI)**^**1**^	
			**(n = 102)**				
Age of source case (n = 219)	16–25	55	31 (56.4)	1	0.06	0.67 (0.45–1.00)	0.05^2^
	26–35	86	40 (46.5)	0.67 (0.34–1.33)			
					0.02^2^		
	>35	78	28 (35.9)	0.43 (0.21–0.88)			
Gender of source case (n = 226)	Female	138	62 (44.9)	1	0.93		
	Male	88	39 (43.3)	0.98 (0.57–1.67)			
Smoking status of source case (n = 225)	Non-smoker	130	52 (40.0)	1	0.09		
	Smoker	95	49 (51.6)	1.60 (0.94–2.73)			
Alcohol use by source case (n = 225)^3^	Never drinks	166	65 (39.2)	1	0.004	1	0.007
	Drinks alcohol	59	36 (61.0)	2.43 (1.32–4.47)		2.59 (1.29–5.22)	
Smear result of source case (n = 224)	Negative	28	9 (32.1)	1	0.17		
	Positive	196	90 (45.9)	1.79 (0.77–4.16)			
Smear grade of source case (n = 217)	Negative	28	9 (32.1)	1	0.13		
	Scanty	18	8 (44.4)	1.69 (0.50–5.73)			
	1+	28	15 (53.6)	2.44 (0.82–7.22)			
	2+	107	44 (41.1)	1.47 (0.61–3.56)			
	3+	36	22 (61.1)	3.31 (1.17–9.37)			
HIV status of source case (n = 224)	Negative	150	79 (52.7)	1	<0.001	1	0.06
	Positive	74	20 (27.0)	0.33 (0.18–0.61)		0.48 (0.22–1.04)	
CD4 count of source case if HIV-positive (n = 71)	<200	33	7 (21.2)	1	0.46		
	>200	38	11 (28.9)	1.51 (0.50–4.56)			
Relationship of source case to child	Parents	100	38 (38.0)	1	0.34		
	Grandparent	31	15 (48.4)	1.53 (0.68–3.45)			
	Uncle or aunt	66	33 (50.0)	1.63 (0.87–3.06)			
	Other	31	16 (51.6)	1.74 (0.77–3.92)			
Duration of exposure between source case and child	Less than a month	30	13 (43.3)	1	0.62		
	One month to six months	92	38 (41.3)	0.92 (0.40–2.11)			
	More than six months	106	51 (48.1)	1.21 (0.54–2.74)			
Primary caregiver to child	Index case	55	21 (38.2)	1	0.26		
	Not index case	173	81 (46.8)	1.43 (0.77–2.65)			
Frequency of contact between source case and child	Daily	213	93 (43.7)	1	0.23		
	Less frequently	15	9 (60.0)	1.94 (0.67–5.63)			
Intensity of contact between child and source case	Sleeps in the same bed	57	25 (43.9)	1	0.67		
	Sleeps in the same room	34	13 (38.2)	0.79 (0.33–1.89)			
	Sleeps in the same house	101	45 (44.6)	1.03 (0.53–1.98)			
	Sleeps in a different house	36	19 (52.8)	1.43 (0.62–3.31)			

^1^Adjusted for ethnicity, age of child, previous TB in the child, age of source case, alcohol use in source case and HIV status of source case, ^2^Test of trend, ^3^Regular alcohol use in source case as reported by the parent or legal guardian of the child, *AOR* adjusted odds ratio, *CI* confidence interval.

### Risk factors for prevalent TB disease

Younger age of the child was associated with increased odds of disease (p-value for test of trend; p = 0.01) as was HIV-positive status in the child (AOR: 25.3; 95% CI: 1.63-393; p = 0.01), HIV positivity of the source case (AOR: 4.07; 95% CI: 1.19-13.8; p = 0.03), increasing number of rooms in the house (AOR: 1.39; 1.02-1.91; p = 0.04) and alcohol use by the source case (AOR 2.90; 95% CI 0.90-9.31; p = 0.07). Male gender (AOR: 0.29; 95% CI: 0.08-1.00; p = 0.04) was associated with reduced odds of disease. See Tables 3 and 4.

**Table 3:** Risk factors for disease in child contacts of multidrug-resistant tuberculosis: child and household characteristics (n = 102)

**Variable**		**Total**	**Disease**	**OR**	**p-value**	**Age-adjusted OR**	**p-value**
		**(n = 102)**	**(row %)**	**(95% CI)**		**(95% CI)**	
			**(n = 15)**				
Age (n = 100)	<1 year	15	4 (26.7)	1	0.28	n/a	n/a
	1–2 years	15	3 (20.0)	0.69 (0.12–3.79)			
	2–3 years	25	6 (24.0)	0.87 (0.20–3.77)			
	3–4 years	27	2 (7.4)	0.22 (0.03–1.38)	0.01^1^		
	4–5 years	18	0 (0)	-			
Gender	Female	46	11 (23.9)	1	0.02	1	0.04
	Male	56	4 (7.1)	0.24 (0.72–0.83)		0.29 (0.08–1.00)	
Ethnicity	Xhosa	30	7 (23.3)	1	0.13		
	Colored	70	8 (11.4)	0.42 (0.14–1.33)			
Previous TB treatment	No	86	14 (16.3)	1	0.32		
	Yes	16	1 (6.3)	0.34 (0.04–2.81)			
HIV status (n = 99)	Negative	96	13 (13.5)	1	0.04	1	0.01
	Positive	3	2 (66.7)	12.8 (1.07–151.1)		25.3 (1.63–393)	
Weight-for-age z-score (n = 97)	More than −1	60	10 (16.7)	1	0.19		
	−1 to −2	26	1 (3.8)	0.20 (0.02–1.65)			
	Less than −2	11	1 (9.1)	0.50 (0.06–4.45)			
BCG scar visible (n = 101)	No	14	2 (14.3)	1	0.95		
	Yes	87	13 (14.9)	1.05 (0.21–5.27)			
Type of residence (n = 98)	Tin shack	8	1 (12.5)	1	0.84		
	Brick house	79	12 (15.2)	1.25 (0.14–11.1)			
	Wendy house	11	1 (9.1)	0.70 (0.04–13.2)			
Number of rooms in house	1-2	28	2 (7.1)		0.04	1.39 (1.02–1.91)	0.04^1^
	3-4	42	4 (9.5)	1.37 (0.23–8.03)			
	>4	32	9 (28.1)	5.09 (1.00–26.0)	0.02^1^		
Number of people living in house	≤5 people	47	5 (10.6)	1	0.29		
	>5 people	55	10 (18.2)	1.87 (0.59–5.91)			
Density of people living in house	≤2 people per room	72	12 (16.7)	1	0.39		
	>2 people per room	30	3 (10)	0.56 (0.14–2.16)			
Water source (n = 100)	House tap	84	12 (14.3)	1	0.65		
	No house tap	16	3 (18.8)	1.38 (0.34–5.60)			
Toilet	Flush toilet in house	79	12 (15.2)	1	0.80		
	Other	23	3 (13.0)	0.84 (0.21–3.26)			

^1^Test of trend, *OR* odds ratio, *CI* confidence interval.

**Table 4:** Risk factors for disease in child contacts of multidrug-resistant tuberculosis: source case and exposure characteristics (n = 102)

**Variable**		**Total**	**Disease**	**OR**	**p-value**	**Age-adjusted OR**	**p-value**
		**(n = 102)**	**(row %)**	**(95% CI)**		**(95%CI)**	
			**(n = 15)**				
Age of source case (n = 99)	16–25	31	4 (12.9)	1	0.81		
	26–35	40	5 (12.5)	0.96 (0.24–3.94)			
	>35	28	5 (17.9)	1.47 (0.35–6.12)			
Gender of source case (n = 101)	Female	62	11 (17.7)	1	0.31		
	Male	39	4 (10.3)	0.53 (0.16–1.80)			
Smoking status of source case (n = 101)	Non-smoker	52	8 (15.4)	1	0.88		
	Smoker	49	7 (14.3)	0.92 (0.31–2.75)			
Alcohol use by source case (n = 101)^1^	Never drinks	65	6 (9.2)	1	0.04	1	0.07
	Drinks alcohol	36	9 (25.0)	3.28 (1.06–10.1)		2.90 (0.90–9.31)	
Smear result of source case (n = 99)	Negative	9	0 (0)	-	0.35		
	Positive	90	15 (16.7)	-			
Smear grade of source case (n = 98)	Negative	9	0 (0)	-	0.05		
	Scanty	8	0 (0)	-			
	1+	15	5 (33.3)	-			
	2+	44	9 (20.5)	-			
	3+	22	1 (4.5)	-			
HIV status of source case (n = 99)	Negative	79	8 (10.1)	1	0.009	1	0.03
	Positive	20	7 (35.0)	4.78 (1.45–15.5)		4.07 (1.19–13.8)	
CD4 count of source case if HIV-positive (n = 18)	<200	7	3 (42.9)	1	0.78		
	>200	11	4 (36.4)	0.76 (0.11–5.28)			
Relationship of source case to child	Parents	38	5 (13.2)	1	0.62		
	Grandparent	15	4 (26.7)	2.4 (0.55–10.6)			
	Uncle or aunt	33	4 (12.1)	0.91 (0.22–3.71)			
	Other	16	2 (12.5)	0.94 (0.16–5.45)			
Duration of exposure between source case and child	Less than a month	13	3 (23.1)	1	0.35		
	One month to six months	38	7 (18.4)	0.75 (0.16–3.47)			
	More than six months	51	5 (9.8)	0.36 (0.07–1.77)			
Primary caregiver to child	Index case	21	2 (9.5)	1	0.46		
	Not index case	81	13 (16.0)	1.82 (0.38–8.76)			
Frequency of contact between source case and child	Daily	93	12 (12.9)	1	0.12		
	Less frequently	9	3 (33.3)	3.38 (0.74–15.3)			
Intensity of contact between child and source case	Sleeps in the same bed	25	3 (12.0)	1	0.47		
	Sleeps in the same room	13	1 (7.7)	0.61 (0.06–6.54)			
	Sleeps in the same house	45	6 (13.3)	1.13 (0.26–4.96)			
	Sleeps in a different house	19	5 (26.3)	2.62 (0.54–12.7)			

^1^Regular alcohol use in source case as reported by the parent or legal guardian of the child, *OR* odds ratio, *CI* confidence interval.

### Relationships between exposures

HIV positivity in the source case was associated with HIV-positive status in the child (p = 0.019), but also with ethnicity (p < 0.001), and with the age of the source case (p-value for test of trend = 0.003). Ethnicity of the child was associated with type of residence (p < 0.001), type of water source (p < 0.001) and type of toileting (p < 0.001). The age of the source case was associated with sputum smear status (p = 0.001), with older source cases less likely to have sputum smear-positive TB.

## Discussion

This is the first study, to our knowledge, to assess risk factors for *M. tuberculosis* infection and disease in children exposed to an adult with MDR-TB. Consistent with data from the natural history of drug-susceptible TB in children, we demonstrate that as children become older, they are more likely to be infected but once infected, that younger children are more likely to develop disease. We also show that HIV-positive children, while having no additional risk of *M. tuberculosis* infection, have a substantially increased risk for disease following infection. The proportion of children who were HIV-positive in our study was relatively low consistent with ongoing household studies of drug-susceptible disease in the same communities (personal communication: Anneke Hesseling) and published household contact investigations undertaken previously in this setting [7]. This likely reflects effective prevention of mother-to-child HIV transmission programs in the Western Cape. Alcohol use in the source case appears to be a risk factor for infection in the child and possibly for disease. The relationship between ethnicity, HIV positivity, age and sputum-smear status of the source case is complex. In our study, older source cases were more likely to be HIV-positive and ethnicity was strongly associated with HIV positivity. Other studies have demonstrated that HIV-positive adults with pulmonary TB are less frequently smear-positive than HIV-negative adults [42]. The increased risk of TB disease in child contacts of HIV-positive source cases may be due to biological (e.g. increased risk of HIV positivity) and epidemiological factors in HIV-affected households.

Previous studies have demonstrated that as children get older they are more likely to become infected, likely due to increasing duration of exposure to more potential source cases and more interaction with the community in addition to household exposure [9]. The relationship between alcohol use in the source case and both infection and disease in the child requires further study. It may be that alcohol is a surrogate for other socioeconomic factors but alcohol was not associated with ethnicity or any of the other socioeconomic exposures recorded in our study. It may be that source cases that drink are in some way more infectious than those who do not drink or that their behavior is more likely to lead to infection (e.g. prolonged exposure due to diagnostic and treatment delay) in the child [43]. Adherence to treatment may be affected as might health-seeking behavior. Alcohol is a demonstrated risk factor for TB disease in adults with drug-susceptible [44] and MDR-TB [45]. That HIV positivity [31] and younger age [27] of the child is associated with increased risk of progression to disease, following infection, is well described in the drug-susceptible TB literature, and we have demonstrated that it also appears to be true for children exposed to MDR-TB. The proportion of children with disease is consistent with other household contact studies from this setting [7]. The association between gender of the child and disease also requires further examination. It is possible that girls are more likely to progress to disease or this association may reflect some sociological or cultural attitude to child-rearing or health-seeking behavior. Houses with more rooms may be a surrogate for socioeconomic status, TB transmission, lifestyle, behavior or nutrition. It may be that multiple families live in houses with more rooms, whereas buildings with fewer rooms only house one family.

A limitation of our study is the relatively small number of children included, and the small number of children with disease. This may have concealed associations that may have been evident if larger numbers had been included. In previous drug-susceptible childhood contact studies, measures of intensity (e.g. proximity of sleeping) and duration of exposure demonstrated a graded relationship with risk of infection in the child, as did the infectiousness of the source case [9,46]. We were unable to show such relationships. We also did not compare children exposed to MDR-TB with children exposed to drug-susceptible TB to determine if systematic differences between the two populations exist (e.g. in child and source case demographics or risk factors for infection and disease). It is possible that MDR-TB in the source case, where typically long exposure durations are seen due to previous failed first-line treatments, may potentially obscure the relationship between risk factors and infection. It would also have been useful to compare MDR-TB-exposed children with community controls without a known source case to document the background (i.e. community, presumably drug-susceptible) infection rate. A further limitation is the study design; although risk factors for the different states (exposure, infection and disease) were evaluated, it was not possible to determine whether the child or the identified source case acquired the infection/disease first. Finally, the definition of infection in our study was one TST measurement undertaken at the initial evaluation. Not only is TST an imperfect measure of infection, but we also did not complete TST retesting at follow-up visits in this cross-sectional study.

## Conclusions

Our study has several implications. Ongoing vigilance should be used in the clinical evaluation of child household contacts, particularly very young (less than three years) or HIV-positive children, following exposure to an MDR-TB source case, based on the high risk of disease progression. In the light of the high demonstrated risk of *M. tuberculosis* infection and of disease progression, the need for contact investigations following an MDR-TB diagnosis in the source case is emphasized, as is the need to carry out these investigations rapidly so that children are identified before they have progressed to disease. Alcohol dependency programs must be integrated into MDR-TB programs given the close relationship between the two epidemics. Finally, the implications of ethnicity and socioeconomic factors on the risk of infection and disease in children require further evaluation.

## Abbreviations

AOR: Adjusted odds ratio; cART: Combination antiretroviral therapy; CI: Confidence Interval; DST: Drug susceptibility test; HIV: Human immunodeficiency virus; IGRA: Interferon-gamma release assay; IQR: Inter-quartile range; LRT: Likelihood ratio test; MDR: Multidrug-resistant; OR: Odds ratio; TB: Tuberculosis; TCH: Tygerberg children’s hospital; TST: Tuberculin skin test; WHO: World health organization.

## Competing interests

The authors declare that they have no competing interests.

## Authors’ contributions

JAS designed the study, collected and analyzed the data, drafted the initial manuscript and then approved the final version. ACH helped to design the study, revised the manuscript and approved the final version. PGF helped to design the study, revised the manuscript and approved the final version. KF assisted in the data analysis, revised the manuscript and approved the final version. HSS helped to design the study, helped to collect the data, revised the manuscript and approved the final version. All authors read and approved the final manuscript.

## Pre-publication history

The pre-publication history for this paper can be accessed here:

http://www.biomedcentral.com/1471-2334/13/392/prepub
